# MycoKey Round Table Discussions of Future Directions in Research on Chemical Detection Methods, Genetics and Biodiversity of Mycotoxins

**DOI:** 10.3390/toxins10030109

**Published:** 2018-03-01

**Authors:** John F. Leslie, Veronica Lattanzio, Kris Audenaert, Paola Battilani, Jeffrey Cary, Sofia N. Chulze, Sarah De Saeger, Annamaria Gerardino, Petr Karlovsky, Yu-Cai Liao, Chris M. Maragos, Giuseppe Meca, Angel Medina, Antonio Moretti, Gary Munkvold, Giuseppina Mulè, Patrick Njobeh, Ivan Pecorelli, Giancarlo Perrone, Amedeo Pietri, Juan M. Palazzini, Robert H. Proctor, Endang S. Rahayu, Maria L. Ramírez, Robert Samson, Jörg Stroka, Michael Sulyok, Mark Sumarah, Cees Waalwijk, Qi Zhang, Hao Zhang, Antonio F. Logrieco

**Affiliations:** 1 Department of Plant Pathology, Throckmorton Plant Sciences Center, 1712 Claflin Avenue, Kansas State University, Manhattan, KS 66506, USA; jfl@ksu.edu; 2 Institute for the Science of Food Production, National Research Council (ISPA-CNR), via Amendola 122/O, 70126 Bari, Italy; veronica.lattanzio@ispa.cnr.it (V.L.); antonio.moretti@ispa.cnr.it (A.M.); giuseppina.mule@ispa.cnr.it (G.M.); giancarlo.perrone@ispa.cnr.it (G.P.); 3 Laboratory of Applied Mycology and Phenomics, Faculty of Bioscience Engineering, Ghent University, Valentyn Vaerwyckweg 1, Campus Schoonmeersen—Gebouw C, 9000 Gent, Belgium; kris.audenaert@ugent.be; 4 Department of the Science of Sustainable Vegetable Production, Faculty of Agriculture, Food and Environmental Sciences, Universitá Cattolica del Sacro Cuore, via E. Parmense, 84-29122 Piacenza, Italy; paola.battilani@unicatt.it; 5 Food and Feed Safety Research, Southern Regional Research Center, USDA-ARS, 1100 Robert E. Lee Boulevard, New Orleans, LA 70124, USA; jeff.cary@ars.usda.gov; 6 Departamento de Microbiología e Immunología, Facultad de Ciencias Exactas Físico-Químicas y Naturales, Universidad Nacional de Río Cuarto, Rutas 8 y 36, Km 601, Río Cuarto 5800, Córdoba, Argentina; schulze@exa.unrc.edu.ar; 7 Department of Bio-analysis, Faculty of Pharmaceutical Sciences, Ottergemsesteenweg 460, Ghent University, 9000 Gent, Belgium; sarah.desaeger@ugent.be; 8 Institute of Photonics and Nanotechnology, National Research Council (CNR-IFN), via Cineto Romano 42, 00156 Rome, Italy; annamaria.gerardino@ifn.cnr.it; 9 Molecular Phytopathology and Mycotoxin Research, University of Goettingen, Grisebachstrasse 6, D-37077 Goettingen, Germany; pkarlov@gwdg.de; 10 Molecular Biotechnology Laboratory of Triticeae Crops, College of Plant Science and Technology, Huazhong Agricultural University, Shizishan Street 1, Hongshan District, Wuhan 430070, China; yucailiao@mail.hzau.edu.cn; 11 Mycotoxin Prevention and Applied Microbiology, National Center for Agricultural Utilization Research, USDA-ARS, 1815 N. University Street, Peoria, IL 61604, USA; chris.maragos@ars.usda.gov; 12 Laboratory of Food Toxicology, Department of Preventive Medicine, Nutrition and Food Science Area, Faculty of Pharmacy, University of Valencia Avenida Vicent Andres Estelles s/n, 46100 Burjassot, Valencia, Spain; giuseppe.meca@uv.es; 13 Applied Mycology Group, Cranfield Soil and Agri-Food Institute, Cranfield University, College Road, Cranfield MK43 0AL, UK; a.medinavaya@cranfield.ac.uk; 14 Department of Plant Pathology and Microbiology, Iowa State University, 160 Seed Science Center, Ames, IA 50011, USA; munkvold@iastate.edu; 15 Department of Biotechnology and Food Technology, University of Johannesburg, P.O. Box 17011, Doornfontein Campus, Gauteng 2028, South Africa; pnjobeh@uj.ac.za; 16 Environmental Contaminants Laboratory, Istituto Zooprofilattico Sperimentale Umbria e Marche (IZSUM), via G. Salvemini 1, 06126 Perugia, Italy; i.pecorelli@izsum.it; 17 Institute for the Science of Food Production, National Research Council (ISPA-CNR), via Amendola 122/O, 70126 Bari, Italy; giancarlo.perrone@ispa.cnr.it; 18 Departamento de Microbiología e Immunología, Facultad de Ciencias Exactas Físico-Químicas y Naturales, Universidad Nacional de Río Cuarto, Rutas 8 y 36, Km 601, Río Cuarto 5800, Córdoba, Argentina; jpalazzini@exa.unrc.edu.ar; 19 Mycotoxin Prevention and Applied Microbiology, National Center for Agricultural Utilization Research, USDA-ARS, 1815 N. University Street, Peoria, IL 61604, USA; robert.proctor@ars.usda.gov; 20 Department of Food Technology and Agricultural Products, Universiti Gadjah Mada, Yogyakarta 55281, Indonesia; endangsrahayu@ugm.ac.id; 21 Departamento de Microbiología e Immunología, Facultad de Ciencias Exactas Físico-Químicas y Naturales, Universidad Nacional de Río Cuarto, Rutas 8 y 36, Km 601, 5800 Río Cuarto, Córdoba, Argentina; mramirez@exa.unrc.edu.ar; 22 Westerdijk Fungal Biodiversity Institute, Uppsalalaan 8, 3584 CT Utrecht, The Netherlands; r.samson@westerdijkinstitute.nl; 23 European Union Reference Laboratory for Mycotoxins, European Commission, Joint Research Centre, Directorate F—Health, Consumers and Reference Materials, Retieseweg 111, B-2440 Geel, Belgium; joerg.stroka@ec.europa.eu; 24 Center for Analytical Chemistry, Department of Agrobiotechnology (IFA-Tulln), University of Natural Resources & Life Sciences—Vienna (BOKU), Konrad Lorenzstrasse 20, A-3430 Tulln, Austria; michael.sulyok@boku.ac.at; 25 London Research and Development Centre, Agriculture & Agri-Food Canada, 1391 Sandford Street, London, ON N5V 4T3, Canada; mark.sumarah@agr.gc.ca; 26 Biointeractions and Plant Health, Wageningen Plant Research, Wageningen University, Droevendaalsesteeg 1, 6708PB Wageningen, The Netherlands; cees.waalwijk@wur.nl; 27 Oil Crops Research Institute, Chinese Academy of Agricultural Sciences, Xudong Second Road, Wuhan 430062, China; zhangqi521x@126.com; 28 State Key Laboratory for Biology of Plant Diseases and Insect Pests, Institute of Plant Protection, Chinese Academy of Agricultural Sciences, No. 2 West Yuanmingyuan Road, Beijing 100193, China; zhanghao@caas.cn

**Keywords:** antibodies, biological control, communication with non-scientists, metabolomics, microbiome, multi-mycotoxin detection protocols, nominal group discussion technique, proteomics, transcriptomics, A better understanding of metabolomics from the cellular to the ecosystem level is needed to inform and control mycotoxin production, control and remediation. Antibody-based diagnostics have become an acceptable standard in many practical applications, but sophisticated multi-mycotoxin detection protocols are the future for many official regulatory controls, especially as the number of toxins that are regulated increases and need more standardization and cross-laboratory validation.

## Abstract

MycoKey, an EU-funded Horizon 2020 project, includes a series of “Roundtable Discussions” to gather information on trending research areas in the field of mycotoxicology. This paper includes summaries of the Roundtable Discussions on Chemical Detection and Monitoring of mycotoxins and on the role of genetics and biodiversity in mycotoxin production. Discussions were managed by using the nominal group discussion technique, which generates numerous ideas and provides a ranking for those identified as the most important. Four questions were posed for each research area, as well as two questions that were common to both discussions. Test kits, usually antibody based, were one major focus of the discussions at the Chemical Detection and Monitoring roundtable because of their many favorable features, e.g., cost, speed and ease of use. The second area of focus for this roundtable was multi-mycotoxin detection protocols and the challenges still to be met to enable these protocols to become methods of choice for regulated mycotoxins. For the genetic and biodiversity group, both the depth and the breadth of trending research areas were notable. For some areas, e.g., microbiome studies, the suggested research questions were primarily of a descriptive nature. In other areas, multiple experimental approaches, e.g., transcriptomics, proteomics, RNAi and gene deletions, are needed to understand the regulation of toxin production and mechanisms underlying successful biological controls. Answers to the research questions will provide starting points for developing acceptable prevention and remediation processes. Forging a partnership between scientists and appropriately-placed communications experts was recognized by both groups as an essential step to communicating risks, while retaining overall confidence in the safety of the food supply and the integrity of the food production chain.

## 1. Introduction

Scientific fields are always changing, and it is important to determine the directions in which they are heading as plans are made for future research activities and the translation of research results into applications and policies. MycoKey [1] is an EU-funded Horizon 2020 project whose goal is to generate innovative, integrated, critical solutions that enable stakeholders to effectively and sustainably manage mycotoxins along both food and feed chains. The project takes a multi-disciplinary approach and includes participants and institutions from multiple countries who implement tools based on open innovation, and disseminate research outputs to government, commercial, and academic audiences for the improvement of food safety and security. MycoKey is a follow-up to the MycoGlobe and MycoRed EU programs that resulted in important publications on mycotoxins in both developed and developing country contexts [2,3].

This wide-ranging approach is essential when working with the complex, interdisciplinary problem of mycotoxin contamination at European and global levels, and to transfer practical solutions to organizations and individuals that participate in modern food chains. MycoKey includes a series of “Roundtable Discussions” to gather information from leading researchers, both within and outside the project, on directions in which the field of mycotoxicology is trending. The first two roundtables were held immediately following the first MycoKey International Conference in Ghent, Belgium in September 2017. One of the roundtables focused on advances in chemical detection and monitoring of mycotoxins that are regulated [4] and emerging [5], and the other on questions of genetics and biodiversity as they relate to mycotoxin production. The results from these discussions and their significance are summarized in this report. Other Roundtable Discussions sponsored by the MycoKey project will focus on (i) predictive modelling of the growth and spread of mycotoxigenic fungi and the production of mycotoxins and (ii) toxicity and regulation of emerging and modified mycotoxins, and will be held at a future date.

## 2. Results

For each Roundtable, a set of four questions was developed by MycoKey project leaders to serve as the focus for the discipline-specific discussions (Table 1). Two additional questions were developed as common questions to be addressed by both groups.

### 2.1. Chemical Detection and Monitoring Discussions

The questions for these discussions related to methods that hold promise for development/improvement in the future. They also explored critical elements in determining where methods can be effectively applied: industry, developing countries and in official control settings. The goal was to identify key elements that could impact when and how methods are used to identify mycotoxins and to increase food safety.

#### 2.1.1. Question CDM 1

In response to CDM Question 1 on methods innovations (Table 2), both groups identified various technologies of interest. The largest number of people had identification of improved extraction methods and the use of non-organic solvents, usually water, on their top-five list. Extraction with non-organic solvents is especially important for many of the kits developed for rapid detection because these kits often are used by individuals who are not familiar with the purchase, storage, recycling and disposal of the organic solvents commonly used in more research-oriented laboratories. A number of other responses focused on improving kits for more general use to improve food safety. These included developing new lateral-flow devices and replacements for existing antibodies and labels, as well as general improvements to existing kits in terms of specificity, reliability, and quantitative as well as qualitative detection capabilities. Price was identified as an important property of these kits that will impact when, how and how often they will be used. Mechanisms to communicate results through smart phones and on-line data transfers to enable broader, more rapid sharing of results also will increase the value of the data obtained from more widespread use of test kits.

Improved technology also was needed to improve physical detection protocols. Further development of multi-mycotoxin methodologies had the highest priority score of all topics. Improving the sensitivity/robustness of and standards available for MS (Mass Spectrometry) work, miniaturization of MS equipment to make it more portable, and development of a reliable database that could be used with emerging mycotoxins were identified as important needs for the field to advance. Other physical methods where significant progress was thought possible include FT-NIR and FT-MIR technology with a database to help interpret the results obtained.

#### 2.1.2. Question CDM 2

CDM Question 2 asked for critical elements in a method that make it suitable for implementation in industry (Table 3). The common responses from both groups were for methods that were cost-effective, easy to use, quick, reliable and suitable for automation. Other desired method properties included safe handling, easy calibration/verification/quality control, and traceability of the process. Sensitivity and wide applicability also were desirable characters, but it was not clear that improvements in both of these characters could be made in the same process simultaneously.

Other responses focused not on the methods themselves, but on how materials/samples to be tested are acquired or on data management after results have been obtained. Sampling remains the single largest variable in mycotoxin assays due to irregular distribution of toxins within a sample. Thus, development of and training in the use of standardized sampling protocols are essential, even though there remains no substitute for screening a large, homogenized sample. Securing a sustainable supply of materials with which to conduct assays and inter-laboratory trials for validation that enable compliance with regulations/standards and fit within a company’s culture are all critical for a method to be of use over the long term. Managing the data obtained from mycotoxin assays also is critical. Ensuring that data can be quickly and easily interpreted and made available to all who need it in a timely manner is critical. Interpreting data in the light of the sampling plan that was used to gather it becomes easier if there are standard plans with set conditions that have been validated for statistical reliability.

#### 2.1.3. Question CDM 3

CDM Question 3 on methods suitable for developing countries had the fewest responses of any of the questions posed (Table 4). All of the responses seemed focused on antibody-based tests in some form, and physical tests such as MS or FT-NIR were not specifically mentioned and apparently were not considered to be practical for widespread use in developing countries. Three of the consensus responses to this question were the same as those for CDM2 for industrial usage: a simple, validated inexpensive method is desired. For developing countries, a test must be ready to use, which was not a priority for the industrial methods and suggests that users in the developed world have sufficient capacity to make at least some alterations to methods as needed. Having on-site support and training available was also thought to be essential for developing country scenarios, which probably recognizes that human and institutional capacity building remains a major limitation in most developing countries. Training at post-secondary levels is needed for individuals to function effectively in programs and laboratories, as is institutional capacity building to provide the infrastructure in which these individuals can work. To compensate for human and institutional shortcomings, the methodology in a developing country scenario will need to be “smarter” and more robust in order to provide functional answers.

Some of the responses identified challenges that are more likely in a developing country situation. Methods that meet these challenges are more likely to be effectively adopted in country. Some of the characters of a readily adoptable method include: (i) a significant shelf life under a range of environmental conditions, e.g., if cold chain storage is not available; (ii) equipment that can function with minimal infrastructure, e.g., without constant power or from batteries that could be charged with solar cells; (iii) methods that needed only water as a solvent; (iv) a stable supply chain for materials used by the method; and (v) ready availability of spare parts for easily repaired equipment. If only the detection of a critical level of mycotoxin contamination is required, then the detection protocol should require little, if any, specialized equipment.

#### 2.1.4. Question CDM 4

This question focused on the evolution of methods for official control, especially as the spectrum of regulated mycotoxins expands and running individual tests for individual toxins becomes less practical when tests are required for numerous toxins. QC of methods for official control becomes critical given that mycotoxins can be an important non-tariff trade barrier and the economic cost of rejecting shipments can be quite high. Three of the six common responses (Table 5) to this question focused on having reference materials with or without toxins as well as stable, isotope-labelled internal standards. None of the other responses focused directly on reference materials, although such materials could be included as part of protocols identified as significant. 

Protocols were a common theme that carried across many responses as well. The other three common responses focused on protocol validation: with labs, test kits and data sets identified as entities for which validation was essential. Labs were also seen as important in terms of staff training, accreditation, and sample management and tracking. In terms of data sets, responses identified criteria for matrix effects and good performance in inter-laboratory multi-mycotoxin tests as important considerations for QC elements in official control processes. Finally, protocols were identified in a number of forms, primarily that they conform to an identifiable standard, but also that they use standardized data, materials, and methodological constraints in the process of determining reported values.

### 2.2. Genetics and Biodiversity Discussions

The questions asked of the genetics and biodiversity Roundtable participants focused on the identification of critical scientific advances that could potentially be exploited to develop novel strategies for the control of mycotoxins. Many mycotoxin biosynthetic pathways are now well understood and yet links between environmental cues or in planta metabolism and mycotoxin production are at best poorly understood. Microbiome studies offer the potential to better understand both mycotoxin production and the impact of mycotoxins in humans and domesticated animals. Answers to questions such as these will form the research agendas for future work on mycotoxin control by identifying biological characters that can be used as targets for control as new controls are designed and deployed.

#### 2.2.1. Question GB 1

This question (Table 6) focused on identifying regulators that are not encoded within the biosynthetic pathway clusters and that affect multiple biosynthetic pathways. The leading response from the combined groups of “comparative studies under different stress conditions” captures the important role of stress associated with most secondary metabolism, and the need for comparative studies, while also leaving large gaps to be filled in terms of particular studies that could or should be conducted. Most of the rest of the responses concentrated on evaluating various types of studies and stresses. Responses common to both groups include systematic knockout of all genes in a fungal species, RNAi and random mutagenesis. It is important to evaluate both loss of function activities, e.g., gene deletions and RNAi, in such screens, as well changes that can result in altered function, e.g., random mutagenesis. Individual group responses continue along these lines with transposon mutagenesis, identification and analysis of gene networks, and inactivating transcription factors in the loss-of-function category, and comparative genomics, evaluating natural variation, and screening existing cDNA libraries and transcriptomic data as approaches to detect traits in which function has been altered. Although there was no particular common point listed by both groups, metabolomics and proteomics were identified as approaches for further study in terms of intermediate and end products produced, and the responses resulting from challenges with various compounds.

#### 2.2.2. Question GB 2

Microbiome studies have emerged as an important new tool for evaluating many traits not obviously connected to a microbial community (Table 7). Studies of biology of field communities, e.g., in soil or in plants, were responses from both groups, as was the composition of human and animal intestinal microbiomes (including the rumen). How environmental and other factors might affect microbiomes also was considered important, including stresses such as: (i) changes as a life/disease cycle progresses; (ii) impact of environmental changes including, fungicides and biocontrols; (iii) availability of disease-resistant host lines; (iv) climate change; and (v) storage conditions. Intentional experimental manipulation of the environment also was suggested in several forms, including: (i) adding probiotics to diets; (ii) adding toxin-producing and toxin-nonproducing strains to existing soil or plant microbiomes; and (iii) adding mycorrhizal fungi to a soil microbiome. All of the responses lead to descriptive answers, i.e., which microbes (and how many) are found when/where. These baseline descriptions are essential groundwork for more sophisticated questions regarding microbiome manipulation to be asked in the future.

#### 2.2.3. Question GB 3

Plant-fungal interactions can influence mycotoxin production, with some interactions well understood and others little more than “black boxes” for which little mechanistic information is available. There was only a single common response to this question (Table 8), i.e., that transcriptomic studies were needed with both toxin-producing and toxin-nonproducing strains. This lack of agreement suggests that many avenues of productive research could be pursued in the process of answering this question. A number of other comparative studies also were suggested, primarily studies in which cultural conditions were varied in some form, e.g., in vitro vs. in planta, stress conditions, competition, and varying host/pathogen combinations. Transcriptomics and metabolomics of both the plant and the fungus were considered important analyses, and with particular items/conditions identified as having greater value, e.g., plant defense mechanisms, metabolism of toxin breakdown products, and disabling genes though knockouts or RNAi. Determining how biocontrol agents and strategies work, identifying genes useful for marker-assisted breeding, and using fungal cultures grown in the presence of particular plant metabolites all represent methods that could provide data that could be used for the practical control of mycotoxin production.

#### 2.2.4. Question GB 4

Question 4 focused on applications that could help reduce/manage mycotoxin production and/or remediate mycotoxin contaminated materials. The three common responses (Table 9) were all quite different from one another, and had additional supporting responses provided by only a single group. The first common response was to implement biocontrol practices, with both atoxigenic fungal strains and bacterial strains mentioned as possibilities. Increasing biodiversity and altering cultural conditions in the field could play a role in the effectiveness of the biocontrols. A second was to use RNAi in GMO host plants. This approach was supported by other responses that suggested altering expression of transcription factors and other gene silencing strategies. The third general area was to identify microbes and enzymes capable of degrading/immobilizing mycotoxins. These solutions might require specially-engineered/-selected organisms or special processing or storage conditions. Another strategy was to manipulate the environment by changing conditions to reduce mycotoxin biosynthesis through altering biodiversity, physiological stress, or the application of novel insights gained from studies of fungal ecology. Crop rotation, resistant plant germplasm, and agronomic studies could guide research and decisions made on environment-altering control strategies. Incorporating substances that suppress expression of mycotoxin biosynthetic genes into the environment and evaluating the impact of chemical control of fungi on toxin production also fall into this general set of control strategies.

### 2.3. Common Questions

Two questions were asked of members of both roundtables. These questions were not discipline specific, but rather were aimed at needs for near-future information for the field of mycotoxicology as a whole and information that needs to be understood by those who are not an immediate part of the mycotoxicology research community. These questions seek responses to identify topics that are critical for public support of research on mycotoxins and their varied impacts across the food chain and human life in general.

#### 2.3.1. Common Question 5

This question has as its focus short-term research needs that can be satisfied before the end of the current funding cycle for the major EU-funded mycotoxin research projects in 2020. Responses ranged from health issues and dietary supplements, to designing biocontrols, and transferring existing technology from the lab to farmers and other stakeholders along the supply chain whose actions affect the actual levels of various mycotoxins in foods and feeds.

There was no response common to all four groups (Table 10). The only response common to three groups was to evaluate the impact of climate change on mycotoxin production, which was supported by single group responses to identify impacts of climate change on predictive models, and to better understand interactions of toxigenic fungi in different ecological settings. This lack of consensus illustrates the complexity of the problem and the myriad different approaches that can be taken to address it.

There were eight responses that were common to two groups. Several of these focused on biological aspects of plant/fungal growth and interaction. One of these eight responses was to better understand host-pathogen interactions, a response that was supported by single group responses to better understand fungal gene regulation and metabolism, to sequence more fungal genomes, to understand pathogen specialization, to develop resistant host plant varieties, and to identify specific targets in pathogens for control. A second was to better understand biocontrols and how they work, a response supported by single group responses to include a diversity of organisms and to study toxins and systems other than *A. flavus* and aflatoxins. Understanding the biodiversity within *A. flavus* atoxigenic strains also was identified as an area for further research, and the possibility of engineering strains for use as biocontrol agents needs further exploration.

A second group of responses focused on various technologies. One response was for integrated technologies for control of plant diseases and mycotoxin production, and the development of SOPs for farming practices to minimize mycotoxin contamination. Other related responses were to move available technology to the field, scale them up and validate their utility, and to determine the adaptability of existing technologies for use in more host/pathogen systems.

Management of information for potential use beyond the mycotoxin research community was expressed in several different ways. For example, sharing information on new methods, their reliability, and their potential roles in controlling movement of contaminated materials, and the problems of detecting all of the significant variant forms of well-known toxins, and other emerging toxins that are less well known but could still be food and feed safety concerns. These efforts could lead to deeper EU-China collaborations, and increase awareness by the general public of mycotoxins, the health and economic problems they are associated with, and the benefits resulting from their control in both developed and developing countries. Analyzing previously-funded EU-mycotoxin projects and developing a database that allows access to documents relevant to mycotoxin research would provide broader context and stronger background for future EU projects that have mycotoxins as a focus.

Finally, there were responses that fell into the areas of determining health risks and the remediation of contaminated foods and feeds. Health risks are the ultimate reason for working on mycotoxins, with concerns about health usually flowing into economic losses through trade reductions and non-utilization of food and feed stuffs for which good economic data are not easy to find, if they are available at all. Health studies need to be expanded beyond the usual focus on a single toxin with death the usual measure of toxicity to include studies with multiple toxins and chronic sub-acute exposures. Risk assessment studies are needed to validate intervention strategies. Management strategies for contaminated materials need more sophisticated development, and the identification of microbes and physical activities that currently degrade toxins will provide targets for enhancement in future research. The use of binders, enzymes and other additives as food or feed supplements to reduce mycotoxin uptake and exposure remains in need of further exploration as do the fates of and risks posed by masked mycotoxins.

#### 2.3.2. Common Question 6

This question focused on information that the current projects should make available to those outside the immediate mycotoxin research community. Again there were no common responses (Table 11) across all four groups, but six responses were common to three groups, and seven more were common to two groups. Most responses focused on providing basic information with messages such as “toxins are natural” but that they have major economic impacts and serious consequences for human and animal health. Risks encountered depend on diet and exposure can be managed as part of effective food safety systems in both developed and developing countries. Relating risks of toxins relative to those of fungicides and the potential for contamination of foods, e.g., “organic” that often are perceived as having a lower risk were needed to make risks “real”. Farmers were singled out for education efforts to help insure that they follow guidelines to help reduce contamination to the greatest extent possible. Finally, it was important to communicate that scientific research in this area is ongoing and in need of continued support to enable further increases in food safety and security. 

## 3. Discussion

The questions asked and the responses received indicate that research on mycotoxins engages a very diverse set of scientists with interdisciplinary interests and dissimilar areas of expertise. The diversity of the participants makes focusing on single topics more difficult, while also raising the significance level of topics for which there was consensus.

Two results stand out from the chemical detection and monitoring responses. Most importantly is the extent to which antibody-based diagnostics have become an acceptable standard in many practical applications. Much of the discussion was on how to improve these kits and to accelerate the detection of mycotoxins in locations where access to a well-equipped analytical chemistry laboratory is neither timely nor cost-effective, if one can be accessed at all. The second point mirrors the first, through the increasing importance of technically highly sophisticated multi-mycotoxin detection protocols. These protocols need more standardization and cross-laboratory validation, but they will be the future for many official regulatory controls, especially as the number of toxins that are regulated increases. Multi-mycotoxin assays are being developed at a large number of locations and international inter-laboratory validation and comparability of results are needed to establish credibility of results across both scientific and regulatory boundaries. Common critical factors for analyses in academic, industrial, and governmental laboratories and developing countries were speed, expense, accuracy/reliability, data management, and, particularly in developing countries, operator training.

From the genetics and biodiversity responses both the depth and diversity of potential research areas are impressive. In many cases, e.g., the microbiome studies, the current questions being asked are primarily descriptive in nature. There are numerous technological approaches to identify critical regulatory circuits and the molecules that participate in them, but designing experiments that provide unequivocal answers remains a challenge. In some cases, using multiple analytical approaches to assess changes in a single physiological event may be required to understand the complexity of the interactions that occur. For example, comparative studies analyzing gene expression through transcriptomics and proteomics at different stress levels could then be interpreted in even finer detail by including near-isogenic fungal strains with gene deletions or plant hosts expressing RNAi gene-silencing constructs. The basic biology underlying some of the observed facts, e.g., biocontrol with atoxigenic strains, needs to be better understood to determine if it is a phenomenon limited to one or a few fungal mycotoxin systems or whether it has a fundamental basis that would enable its generalization to other systems. Efforts to control the impact of mycotoxins usually are focused on methods that prevent toxin synthesis. Yet toxins often are present for reasons, e.g., weather or cropping system, that are not subject to ready control and the question of remediation becomes a critical one. Remediation is particularly important in developing countries. In developed countries, contaminated material can be effectively discarded and destroyed. In developing countries, however, food security problems usually mean that someone will consume even the most contaminated foodstuffs to avoid starvation. Thus, remediation is as important in developing countries as reduction. That the remediation needs to be inexpensive and relatively simple technically only heightens the challenge.

The chemistry and biodiversity groups’ responses overlap most significantly in suggesting that a better understanding of metabolomics from the cellular to the ecosystem level is needed to inform and expand mycotoxicology in its broadest context. How fungi and the secondary metabolites they produce interact to cause plant diseases and to enhance fungal growth remains substantially unknown. The increasing availability of data from multi-mycotoxin assays, which generate more data than can be meaningfully interpreted, requires new computing algorithms to be adequately explored, and the metabolic profiles available now can help determine if particular genera are present in a sample without doing any culturing or DNA sequencing. The matching of metabolomics with genomics will generate a broader and deeper understanding of the diverse capabilities of these fungi and the ecosystems they inhabit than is currently available. Mycotoxicology, through its inherent interdisciplinary nature, is well positioned to foster such interactions and to benefit from the resulting insights.

The single largest challenge facing the mycotoxin research community probably is one of communication with a broader public. The message being given can be very alarming and, if phrased improperly, can result in communal panic with agricultural commodities such as grains or milk being dumped needlessly in efforts to make the food supply safer. Communication strategies to educate government policy makers and the general public about the risks associated with mycotoxins should be essential components of future iterations of large projects such as MycoKey. Without a knowledgeable audience to receive the results of the work currently being done, enabling the changes needed to further increase food safety and security by reducing mycotoxin exposure will be difficult. Forging partnerships between scientists and well-informed communication experts is essential to ensure that the general public retains confidence in the safety of their food supply. Recently, a group of scientists has formulated the MycoTox Charter [6] to identify critical facts and potential obligations and actions that can be taken to help reduce problems associated with mycotoxins in both developed and developing countries.

## 4. Methods

Roundtable participants were selected by MycoKey project leaders. Participants in the Chemical Detection and Monitoring (CDM) roundtable included: Sarah De Saeger, Annamaria Gerardino, Veronica Lattanzio, Chris Maragos, Giuseppe Meca, Patrick Njobeh, Ivan Pecorelli, Amedeo Pietri, Jörg Stroka, Michael Sulyok, Mark Sumarah, and Qi Zhang. For the Genetics and Biodiversity (GB) roundtable the participants included: Kris Audenaert, Paola Battilani, Jeffrey Cary, Sofia N. Chulze, Petr Karlovsky, Yu-Cai Liao, Angel Medina, Antonio Moretti, Gary Munkvold, Giancarlo Perrone, Juan M. Palazzini, Robert Proctor, Endang S. Rahayu, Maria Laura Ramirez, Robert Samson, Cees Waalwijk, and Hao Zhang. Roundtable participants were selected for diversity in research interests and capabilities, geographic origin, economic status of originating country, and reasons for being interested in mycotoxin questions.

Members of each roundtable were divided into two groups in which the discussions were held. Neither group memberships nor discussions overlapped, so that ideas created within a group were unique to that group. A facilitator, who moderated the discussion, and a reporter, who wrote ideas on flip chart, were identified in each group.

The Roundtable discussions were guided through the use of the Nominal Group Technique, a moderated discussion technique that has been used previously to identify priorities for research with mycotoxins [7]. The Nominal Group Technique provides for equal input from all participants and is well-known as a process for generating a large number of ideas, while also providing a mechanism for ranking them. The rankings and the total list of ideas provide a rich and detailed context from which particular ideas and general trends often can be extracted. For each Roundtable, a set of four questions was developed by MycoKey project leaders to serve as the focus for the discipline-specific discussions (Table 1). Two additional questions were developed as common questions to be addressed by both groups.

The Nominal Group Technique results in a structured discussion, with the discussion of each of the questions requiring 40–70 min. Each structured discussion has five stages:

1. Silent generation of ideas: In this step, each individual considers answers to the proposed questions, and writes them down on a sheet of paper to facilitate their inclusion in the subsequent discussion. It is important that participants do not talk with one another during this time so that the ideas generated are their own and are not part of a larger group thinking process.
2. Sharing ideas: With the reporter first and the moderator last, members of the group share one answer at a time as they go around the group. The reporter writes each response on the flip chart as a word or phrase that captures the essence of the response. When a round of responses is complete, another one follows immediately. There is no expectation that each member of the group will provide the same number of responses, and individuals can continue to add items to the group’s list until their personal list is exhausted. Participants may respond in any round when they have an answer to contribute, or pass if they have nothing additional to contribute at that time. The idea sharing ceases when no one in the group has any more ideas to contribute. A common target for these lists is a total of 15–25 responses, with groups that have fewer than 15 usually encouraged to spend some additional time repeating Step 1 to generate more ideas if the first list is too short.
3. Idea explanation: This discussion is moderated by the facilitator, who ensures that all of the ideas are clearly understood by all of the members of the group. The facilitator also is responsible for ensuring that everyone participates in the discussion in a systematic and equal manner. The discussion usually proceeds from the first to the last topic on the flip chart, with each member presented an opportunity to question or offer an opinion on each response. One way to conduct the discussion is to go around the group with each member raising/making one question/comment at a time on a particular response. Responses should be neither judged nor critiqued, and the process should be emotionally neutral. The reporter may make modifications/clarifications on the flip chart as needed. Ideas may be grouped if the participants who suggested them (not the group as a whole) agree that they are the same. The group also may add new responses to the list.
4. Voting and ranking: Once the ideas have been adequately explained, each participant is given a 3 × 5 index card, or similarly-sized piece of heavy paper. The question number and group name are written on every card. Each participant ranks the five most important answers for the question on the flip chart list, with the most important answer being given a “5”. The second choice answer receives a “4”, the next a “3”, and so on. Each individual must rank five answers on their written list. This process is done silently as the goal is to record individual preferences and not to reach a consensus, although a consensus may be observed when the numbers are tallied. The written lists are returned to the reporter who records the individual ranks next to each response on the flip chart. The flip chart paper and the 3 × 5 cards with the rankings are turned into the session organizers for further analysis.
5. Presentation of results: Results from the discussions are presented on a question by question basis in Table 2, Table 3, Table 4, Table 5, Table 6, Table 7, Table 8, Table 9, Table 10 and Table 11. Within each table, responses that were given by more than four groups are given first and followed by those given by three groups, then two groups and then single groups. Within each of these sets of responses, the responses are ordered based on the number of individuals giving a response, and then by the summed weight of the responses. Responses given in a group, but not on any individual’s list of the top five (so no weighted score), are denoted with a “●” to enable distinction between an unweighted response and a response that was absent, which is denoted with a dash. The wording used for an item is usually the exact wording that was used by the group making the response. When multiple groups gave the same response, the wording was altered, if necessary, to include the common idea expressed by the different groups.

## Figures and Tables

**Table 1:** Questions for discussion at MycoKey Roundtables on chemical detection and monitoring and on genetics and biodiversity.

Number	Question
	Chemical Detection and Monitoring (CDM)
CDM 1	Identify scientific and technological innovations that are promising for method development and implementation. Consider reliability, applicability to real world samples, transferability to end users, etc.
CDM 2	Identify the key elements that make a method suitable for implementation in industry (large companies and SMEs (Small and Medium) enterprises) for auto control, HACCP (Hazard Analysis and Critical Control Points) plans and process management.
CDM 3	Identify key elements that make a method suitable to be implemented in developing countries with poor resources and limited analytical capabilities.
CDM 4	Methods for official control purposes are becoming more heavily reliant on multi-mycotoxin analyses. Identify current Quality Control (QC) procedures and/or metrological tools that are efficient for official control and accredited methods.
	Genetics and Biodiversity (GB)
GB 1	How can cellular signals/regulators/metabolites that alter mycotoxin production/stability, but are not associated with an individual mycotoxin or cluster, be identified and/or characterized?
GB 2	What microbiome studies are needed to understand the importance and impact of mycotoxins (in native and agricultural ecosystems, human and animal health, etc.)?
GB 3	Plant/fungal interactions that increase/decrease mycotoxin biosynthesis occur. How should these interactions be identified/characterized/exploited?
GB 4	How can knowledge of fungal gene regulation and biodiversity be applied to the reduction/management of mycotoxin production and the remediation of mycotoxin contaminated materials?
	Common
5	What information should be generated or questions answered now for research in this area to progress after the current project ends in 2020?
6	What do people outside the scientific community need to know about mycotoxins?

**Table 2:** Nominal group responses to CDM Question No. 1: Identify scientific and technological innovations that are promising for method development and implementation. Consider reliability, applicability to real world samples, transferability to end users, etc.

CDM 1		CDM 2		Response
# ^1^	*S*^2^	#	*S*	
3	5	5	16	Non-toxic solvent (organic) extraction and improvement of extraction methods
2	9	5	18	Multi-mycotoxin technologies (including IAC (Immunoaffinity Columns))
2	7	2	5	Smart phone/on-line transfer of results
1	2	1	4	Label-free detection or new labels for lateral flow devices
●^3^	●	3	12	Development of physical methods based on FT-NIR or FT-MIR technology with a database for interpreting results
●	●	2	7	MS QqQ (Mass Spectrometry Triple Quadrupole) with internal (isotopically) labeled standards
●	●	2	4	Chemically synthesized aptamer probes (based on oligonucleotides) and MIPS, to replace antibody probes
4	15	-^4^	-	Miniaturization of MS
3	10	-	-	Reference materials/PT test (Proficiency Test)
3	7	-	-	Improved antibodies, test kits and strips
-	-	3	4	Tool box-like technology for transportability
2	7	-	-	Increased sensitivity/robustness of mass spectrometry
-	-	2	7	Time resolved fluorescent strip method for ppb level analyses
2	5	-	-	Price
1	5	-	-	Association ELISA and app
-	-	1	5	High specificity and affinity recognizers
-	-	1	4	Validated, reliable database for identification of “emerging” mycotoxins by high resolution mass spectrometry
1	2	-	-	LAMP (Loop-mediated isothermal amplification) for detection of fungi
-	-	1	2	Gold nanoparticle-enabled immunoassays (~ppb limit)
-	-	1	1	Non-target screening methods UPLC-MS
-	-	1	1	Microfluidic device exploiting SMART functionalization
1	1	-	-	Alternatives to antibodies
●	●	-	-	Ambient ionization mass spectrometer
-	-	●	●	Vibrational spectroscopy methods as “indicators” for fungal degradation

^1^ Number of participants ranking this response as one of the five most important. ^2^ Weighted priority score, with each voting member ranking their top five topics. Five points assigned to the most important response and one point to the least significant of the important responses. ^3^ This response provided by one or more members of the group when ideas were listed, but was not identified as one of the five most important responses by any member of the group. ^4^ This response not provided by any member of the group.

**Table 3:** Nominal group responses to CDM Question No. 2: Identify the key elements that make a method suitable for implementation in industry (large companies and small and medium enterprises) for auto control, HAACP plans and process management.

CDM 1		CDM 2		Response
# ^1^	*S*^2^	#	*S*	
5	17	3	7	Cost-effectiveness
4	11	4	11	Easy to use: limited expertise and minimal operator manipulations
3	10	3	7	Process automation
3	12	2	5	High degree of reliability/validity
1	5	2	6	Speed
^4^	-	5	17	Full traceability of measurement process and quality control
-	-	5	12	Easy calibration/verification of functionality
-	-	3	11	Safe, easy handling
2	9	-	-	On-line capability
2	8	-	-	Wide applicability
2	5	-	-	Data accessible at any time
2	3	-	-	Sensitivity
2	2	-	-	Fast, simple data interpretation
2	2	-	-	Compliance with regulations/standards
-	-	1	5	Sampling plan
1	4	-	-	Sustainability
1	2	-	-	Fits supply chain
●^3^	●	-	-	Corporate culture
●	●	-	-	Secure supply of materials
●	●	-	-	“Green” technology

^1^ Number of participants ranking this response as one of the five most important. ^2^ Weighted priority score, with each voting member ranking their top five topics. Five points assigned to the most important response and one point to the least significant of the important responses. ^3^ This response provided by one or more members of the group when ideas were listed, but was not identified as one of the five most important responses by any member of the group. ^4^ This response not provided by any member of the group.

**Table 4:** Nominal group responses to CDM Question 3. Identify key elements that make a method suitable to be implemented in developing countries with poor resources and limited analytical capabilities.

CDM 1		CDM 2		Response
# ^1^	*S*^2^	#	*S*	
6	26	2	5	Cost
4	11	4	14	Simple extraction, purification and QC
2	4	4	11	On site and support training from company
2	8	2	8	Analytical performance/validation well-established
●^3^	●	3	6	Ready to use (tool box)
6	16	^4^	-	Robustness/stability
-	-	5	17	Significant shelf life if cold chain unavailable
5	15	-	-	Independent of electricity/infrastructure
-	-	4	6	Equipment is easy to maintain and repair
3	3	-	-	Rapid method
-	-	2	7	Solar power available for instruments
-	-	1	5	Know-how is in the product and not required of the operator
1	4	-	-	Secure supply of required materials
-	-	1	4	Instrument free detection ability
1	3	-	-	Wide applicability
●	●	●	●	Link to smart phone
●	●	-	-	No solvents needed (other than water)
-	-	●	●	Portable

^1^ Number of participants ranking this response as one of the five most important. ^2^ Weighted priority score, with each voting member ranking their top five topics. Five points assigned to the most important response and one point to the least significant of the important responses. ^3^ This response provided by one or more members of the group when ideas were listed, but was not identified as one of the five most important responses by any member of the group. ^4^ This response not provided by any member of the group.

**Table 5:** Nominal group responses to CDM Question 4. Methods for official control purposes are becoming more heavily reliant on multi-mycotoxin analyses. Identify current QC procedures and/or metrological tools that are efficient for official control and accredited methods. Are inter-laboratory validated methods of analysis still needed?

CDM 1		CDM 2		Response
# ^1^	*S*^2^	#	*S*	
4	15	2	3	Internally labelled standards
2	9	3	9	Certified reference materials
2	6	2	4	Inter-laboratory ring trials/PT-tests
1	2	3	9	Blanks
1	2	1	1	Test kit validation programs
●^3^	●	4	11	Measurement uncertainty protocol
^4^	-	4	15	Mandatory participation in multi-mycotoxin ring trials and PT tests with demonstrable good performance
4	12	-	-	Staff training
4	11	-	-	Instrument calibration
-	-	3	6	Validation document similar to “Sante” document for pesticides
2	10	-	-	QC material
-	-	2	10	Criteria for calculating and evaluating LOQ (Limit of Quantificatioon, scientific, not legislative)
2	5	-	-	Standardized sampling protocols, e.g., GIPSA (Grain Inspection, Packers and Stockyards Administration)
2	2	-	-	Laboratory accreditation
2	5	-	-	Standardized protocol list
2	5	-	-	Standardized sample list
1	4	-	-	Following ISO/AOAC protocols
-	-	1	4	Agreement between “performance criteria” and “fitness for purpose” approaches
1	3	-	-	Acceptance criteria for matrix effects
1	2	-	-	Sample tracking
1	2	-	-	Lab management system
●	●	-	-	Normalized retention indices
●	●	-	-	Regulation of validation parameters

^1^ Number of participants ranking this response as one of the five most important. ^2^ Weighted priority score, with each voting member ranking their top five topics. Five points assigned to the most important response and one point to the least significant of the important responses. ^3^ This response provided by one or more members of the group when ideas were listed, but was not identified as one of the five most important responses by any member of the group. ^4^ This response not provided by any member of the group. ISO = International Standard Organization. AOAC = Association of Official Analytical Chemists.

**Table 6:** Nominal Group responses to GB Question 1. How can cellular signals/regulators/ metabolites that alter mycotoxin production/stability, but are not associated with an individual mycotoxin or cluster, be identified and/or characterized?

GB 1		GB 2		Response
# ^1^	*S*^2^	#	*S*	
5	16	6	23	Comparative studies under different stress conditions
2	2	3	4	Systematic knockout of all genes in a producing fungal species
3	8	1	2	Comparative genomics in silico
1	4	2	3	RNAi of stress responsive genes
●^3^	●	4	11	Evaluate random mutations
-^4^	-	7	32	RNA Seq of fungal and plant genes
-	-	7	19	Metabolomics—host-pathogen interactions & other compounds/organisms
6	24	-	-	Saturation mutagenesis by transposon and screening
-	-	6	12	Gene network analysis
5	18	-	-	Prediction and knockout of transcription factor genes
5	14	-	-	Screen expressed cDNA libraries for enhanced mycotoxin production
4	10	-	-	Evaluate already published transcriptomic data
-	-	3	10	Screen metabolites by challenging pathogens with candidate compounds
3	6	-	-	Affinity chromatography of promoter sequences
-	-	1	4	Quantitative PCR
1	3	-	-	Synthetic biology and bioinformatics
●	●	-	-	Evaluation of natural variation
●	●	-	-	Comparative metabolomics
-	-	●	●	Analyze intermediate metabolic products
-	-	●	●	Compare volatile compound profiles
-	-	●	●	Comparative proteomics of strains
-	-	●	●	Evaluate end-product metabolism

^1^ Number of participants ranking this response as one of the five most important. ^2^ Weighted priority score, with each voting member ranking their top five topics. Five points assigned to the most important response and one point to the least significant of the important responses. ^3^ This response provided by one or more members of the group when ideas were listed, but was not identified as one of the five most important responses by any member of the group. ^4^ This response not provided by any member of the group.

**Table 7:** Nominal group responses to GB Question 2. What microbiome studies are needed to understand the importance and impact of mycotoxins (in native and agricultural ecosystems, human and animal health, etc.)?

GB 1		GB 2		Response
# ^1^	*S *^2^	#	*S*	
4	13	5	21	Metagenomics of human and animal intestinal microbes, including rumen
5	16	4	14	Effect of environment/management on microbiome in the soil/plant
4	19	3	10	Transcriptomics of microbial communities of specific crops exposed to mycotoxins
3	12	1	5	Metagenomics of plant microbiome
●^3^	●	5	13	Microbiome changes over life cycle and/or disease cycle
-^4^	-	4	9	Model climate change effects on microbiome composition
-	-	3	8	Probiotic effects on human and animal gut microbiomes
-	-	3	8	Natural fermentation microbiome
-	-	3	7	Effect of biocontrol on plant/soil microbiome
3	6	-	-	Effect of fungicide on soil and plant microbes of treated crops
3	6	-	-	Genome sequencing of microbes in crop microbiome
3	5	-	-	Effect of mycotoxins on microbiome of harvested crops in storage
2	7	-	-	Microbiomes of crop residues and weeds
-	-	2	7	Microbiomes of resistant and susceptible host lines
2	6	-	-	Microbiomes of agricultural field soil
2	5	-	-	Microbiomes of native/non-cultivated plants
2	5	-	-	Microbiome of feed products
1	3	-	-	Microbiome in the air
1	2	-	-	Experimental ecology with microbes from plant microbiome
-	-	1	2	Effects of mycorrhizae on soil microbiome
-	-	1	1	Rhizosphere microbiome following exposure to toxin-producing and toxin-nonproducing strains
●	●	-	-	Effects of mycotoxins on plant endophytes

^1^ Number of participants ranking this response as one of the five most important. ^2^ Weighted priority score, with each voting member ranking their top five topics. Five points assigned to the most important response and one point to the least significant of the important responses. ^3^ This response provided by one or more members of the group when ideas were listed, but was not identified as one of the five most important responses by any member of the group. ^4^ This response not provided by any member of the group.

**Table 8:** Nominal group responses to GB Question 3. Plant/fungal interactions that increase/decrease mycotoxin biosynthesis occur. How should these interactions be identified/characterized/exploited?

GB 1		GB 2		Response
# ^1^	*S*^2^	#	*S*	
6	23	4	15	Transcriptomic analyses of plant-fungal interactions with toxin-producing and toxin non-producing strains
6	18	-^4^	-	RNAi of targeted plant genes to study effects on mycotoxin production
6	17	-	-	Transcriptomics of target genes in plants and fungi under different conditions
-	-	5	18	Comparative studies with different pathogen/host combinations
-	-	5	15	Stress conditions
4	15	-	-	Comparative fungal transcriptomics in planta and in vitro
4	13	-	-	Metabolomics of plant-fungal interactions
-	-	4	11	Characterize plant defense responses
4	6	-	-	Quantify mycotoxin production in culture with different plant components
-	-	4	6	Competition
-	-	3	10	Conducive studies: identify conditions that favor toxin production
-	-	3	10	Gene splicing
-	-	3	9	Marker assisted breeding
-	-	2	8	Biocontrol
-	-	2	7	Detoxification and evaluation of breakdown products
2	6	-	-	Examine genetic variation in plants that affect mycotoxins
2	3	-	-	RNAi study of plants exposed to mycotoxin producing fungi
-	-	2	4	Gene editing to produce novel proteins and metabolites
-	-	2	4	Antagonistic conditions
1	4	-	-	Identify and knock out fungal effectors to determine impact on mycotoxin production
-	-	1	3	Overexpress plant defense genes
-	-	●^3^	●	Symbiotic relationships between plants and microorganisms

^1^ Number of participants ranking this response as one of the five most important. ^2^ Weighted priority score, with each voting member ranking their top five topics. Five points assigned to the most important response and one point to the least significant of the important responses. ^3^ This response provided by one or more members of the group when ideas were listed, but was not identified as one of the five most important responses by any member of the group. ^4^ This response not provided by any member of the group.

**Table 9:** Nominal Group responses to GB Question 4. How can knowledge of fungal gene regulation and biodiversity be applied to the reduction/management of mycotoxin production and the remediation of mycotoxin contaminated materials?

GB 1		GB 2		Response
# ^1^	*S*^2^	#	*S*	
7	28	5	20	Use atoxigenic strains (as a biocontrol)
3	7	8	27	RNAi induced gene silencing
6	21	4	8	Identify microbes and enzymes capable of degrading mycotoxins
2	4	●^3^	●	Competition and interaction with bacteria
-^4^	-	5	16	Agronomic management to promote beneficial organisms
5	12	-	-	Modify environmental conditions to inhibit mycotoxin biosynthetic genes
4	13	-	-	Identify substances that suppress expression of mycotoxin biosynthetic genes
3	11	-	-	Competition between different species
-	-	3	10	Avoid stress (on fungus)
3	8	-	-	Mycotoxin^-^ mutants as a biocontrol strategy
-	-	3	8	Engineered bioremediation/detoxifying organisms
3	7	-	-	Incorporate knowledge of fungal biodiversity into plant breeding programs
2	7	-	-	Develop strategy based on knowledge of fungal ecology
-	-	2	6	Modeling software
-	-	2	6	Impact of chemical control on fungal metabolites
-	-	2	4	Biodiversity dynamics: relationship with toxin production and regulation
2	3	-	-	Use yeast (cell wall) to absorb mycotoxin(s)
-	-	2	3	Crop rotation
-	-	1	4	Fermentation to reduce toxins
-	-	1	4	Biofumigation
-	-	1	3	Down regulation of transcription factors
-	-	1	1	Toxin production by different species on different host cultivars
●	●	-	-	Apply conditions that suppress mycotoxin synthesis to stored products
-	-	●	●	Improved biocontrol
-	-	●	●	Rapid detection for monitoring by PCR or hybridization
-	-	●	●	Spray-Induced Gene Silencing (SIGS)

^1^ Number of participants ranking this response as one of the five most important. ^2^ Weighted priority score, with each voting member ranking their top five topics. Five points assigned to the most important response and one point to the least significant of the important responses. ^3^ This response provided by one or more members of the group when ideas were listed, but was not identified as one of the five most important responses by any member of the group. ^4^ This response not provided by any member of the group.

**Table 10:** Nominal group responses to Common Question 5. What information should be generated or questions answered now for research in this area to progress after the current project ends in 2020?

CDM 1		CDM 2		GB 1		GB 2		Response
# ^1^	*S*^2^	#	*S*	#	*S*	#	*S*		
3	10	-^4^	-	6	25	1	4	Climate change effects on toxigenic organisms and mycotoxin production
-	-	-	-	5	11	4	12	Improved understanding of host-pathogen interactions
5	16	-	-	3	12	-	-	Understand biocontrol effectiveness and mode of action
2	5	-	-	-	-	6	16	Improved modeling/predictive capabilities
4	13	-	-	-	-	3	12	New integrated technologies for disease/mycotoxin prevention/control
2	7	4	6	-	-	-	-	Spreading information/training on quality control/assurance for multi-mycotoxin methods for HAACP and/or official control
1	1	-	-	-	-	2	6	Global knowledge sharing and cooperation
●^3^	●	4	15	-	-	-	-	Protocol for evaluating and comparing performance of rapid methods
2	4	-	-	-	-	●	●	SOPs for farming practices to minimize mycotoxin contamination
-	-	5	24	-	-	-	-	Meta-study on the findings of all previously-funded EU mycotoxin projects
-	-	5	14	-	-	-	-	A database with links to documents relevant for mycotoxin researchers. Access via the MycoKey web site
-	-	-	-	4	13	-	-	Increase knowledge of fungal gene regulation to reduce/prevent mycotoxin production
-	-	-	-	-	-	4	13	Health impacts of combinations/interactions among mycotoxins
-	-	-	-	-	-	4	12	Validation of technology applications in the field
-	-	4	11	-	-	-	-	Simplified, harmonized procedure for measurement uncertainty calculation
-	-	-	-	4	9	-	-	Interactions of toxigenic fungi in different ecological conditions and impact on mycotoxin production
-	-	-	-	-	-	3	9	Detailed economic analysis of impacts of mycotoxins
-	-	-	-	3	8	-	-	Whole genome sequencing of toxigenic fungi at the population level
-	-	-	-	3	8	-	-	Identify sources of mycotoxin detoxification
-	-	-	-	-	-	3	8	Risk assessment of human/animal exposure to validate intervention strategies
-	-	-	-	3	7	-	-	Predict which mycotoxins suppressed in MycoKey will come back in the future
-	-	-	-	-	-	3	7	Effects of climate change on predictive models
2	10	-	-	-	-	-	-	Development/implementation of mobile app
-	-	-	-	-	-	2	8	End-user benefits
-	-	-	-	2	7	-	-	Increase knowledge of toxigenic fungi world wide
2	6	-	-	-	-	-	-	Increased European Union-China cooperation
-	-	-	-	2	6	-	-	CRISPR/CAS protocol to modify toxigenic fungi for biocontrol
-	-	-	-	2	5	-	-	Define correct species for mycotoxin-producing strains
-	-	-	-	-	-	2	5	Adaptability of strategies to other crops/food chains
2	3	-	-	-	-	-	-	Interlaboratory validation beyond state of the art
-	-	2	3	-	-	-	-	Low cost methods: Identification of methods ready for market/large scale production
2	2	-	-	-	-	-	-	Management of contaminated materials
1	5	-	-	-	-	-	-	Green technologies for reducing mycotoxin exposure
-	-	-	-	1	5	-	-	Define biodiversity of atoxigenic *Aspergillus flavus*
1	4	-	-	-	-	-	-	New remediation protocols
1	4	-	-	-	-	-	-	Effectiveness of binders/detoxifiers
-	-	-	-	1	3	-	-	Develop novel biocontrol strategy(ies) using yeasts and/or bacteria
-	-	-	-	-	-	1	3	Smart labeling environmental sensing
-	-	-	-	-	-	1	3	Efficacy of RNAi under field condition
-	-	1	2	-	-	-	-	Identify topics to be addressed
-	-	-	-	-	-	1	2	Specific targets in pathogens
-	-	-	-	1	1	-	-	How to identify/monitor emerging mycotoxins
●	●	-	-	-	-	-	-	Resistant host plant varieties
●	●	-	-	-	-	-	-	Masked/modified mycotoxins
-	-	-	-	●	●	-	-	Identify suppressor(s) of mycotoxin biosynthesis
-	-	-	-	●	●	-	-	Identify biocontrol agents of *Fusarium* head blight worldwide
-	-	-	-	-	-	●	●	Specialization of fungi to host species
-	-	-	-	-	-	●	●	Analysis of the impact of regulations

^1^ Number of participants ranking this response as one of the five most important. ^2^ Weighted priority score, with each voting member ranking their top five topics. Five points assigned to the most important response and one point to the least significant of the important responses. ^3^ This response provided by one or more members of the group when ideas were listed, but was not identified as one of the five most important responses by any member of the group. ^4^ This response not provided by any member of the group.

**Table 11:** Nominal group responses to Common Question 6. What do people outside the scientific community need to know about mycotoxins?

CDM 1		CDM 2		GB 1		GB 2		Response
# ^1^	*S*^2^	#	*S*	#	*S*	#	*S*		
3	10	5	19	8	35	-^4^	-	Good communication of occurrence of and impacts from mycotoxin contamination
3	6	2	5	7	15	-	-	Toxins are “natural”. Organic food may be more contaminated than conventional food.
2	6	2	7	-	-	8	28	Impact on human and animal health
3	5	4	6	-	-	3	11	Economic impact of mycotoxins
1	1	-	-	●^3^	●	6	23	Risk of mycotoxin exposure depends on diet
1	4	-	-	4	7	1	2	Tools/strategies exist for mycotoxin management
5	25	-	-	5	14	-	-	What are mycotoxins?
-	-	2	8	-	-	4	6	Sensitize farmers to the problem of mycotoxins so that they will invest money and effort to generate high quality products
●	●	4	11	-	-	-	-	Things consumers can do themselves, e.g., check for mold, food handling to prevent spoilage
●	●	-	-	3	8	-	-	Non-dietary sources, e.g., indoor air
●	●	-	-	-	-	1	3	Mycotoxins can be identified and analyzed
1	1	-	-	-	-	●	●	Good food safety system needed in developing countries
●	●	-	-	-	-	●	●	Good food safety systems exist in developed countries
-	-	-	-	7	23	-	-	Mycotoxins are a food and feed safety problem
-	-	-	-	4	12	-	-	Mycotoxins are as dangerous as pesticides and heavy-metal residues
-	-	-	-	-	-	4	8	Mycotoxins are an invisible hazard
-	-	3	12	-	-	-	-	Learn how to obtain official information rather than relying on radio, TV, newspapers and social media
-	-	-	-	-	-	3	11	Need for research funding
3	10	-	-	-	-	-	-	Highest dietary risk factor
3	8	-	-	-	-	-	-	Scientists/authorities are working on mycotoxins
-	-	3	7	-	-	-	-	Safe food requires good manufacturing, monitoring and suitable crops
-	-	-	-	-	-	3	7	Proper grain storage practices
-	-	-	-	-	-	2	8	Health risk assessment for each mycotoxin
2	6	-	-	-	-	-	-	Mycotoxin occurrence in different commodities
-	-	-	-	-	-	2	4	Risks can occur at any stage of the food chain
-	-	-	-	-	-	1	4	Factors conducive to mycotoxin occurrence
-	-	-	-	-	-	1	4	Recognition of mycotoxin producing fungi
1	3	-	-	-	-	-	-	Highest prevalence
1	3	-	-	-	-	-	-	They are a historical problem
-	-	-	-	-	-	1	3	Relative risk of mycotoxins compared with other food safety problems
1	2	-	-	-	-	-	-	Problems with food security
-	-	-	-	-	-	1	1	Thermal stability of most mycotoxins
●	●	-	-	-	-	-	-	Problems may shift due to climate change
●	●	-	-	-	-	-	-	Problem is not the moldy food in the refrigerator
-	-	●	●	-	-	-	-	Information on HAACP protocols
-	-	-	-	●	●	-	-	Risks of consuming mycotoxin contaminated food/feed
-	-	-	-	-	-	●	●	Awareness of legislation and regulations
-	-	-	-	-	-	●	●	Presence of fungi does not mean mycotoxins are present
-	-	-	-	-	-	●	●	Impact of climate

^1^ Number of participants ranking this response as one of the five most important. ^2^ Weighted priority score, with each voting member ranking their top five topics. Five points assigned to the most important response and one point to the least significant of the important responses. ^3^ This response provided by one or more members of the group when ideas were listed, but was not identified as one of the five most important responses by any member of the group. ^4^ This response not provided by any member of the group.
